# Successful Management of Extreme Hyperglycemia (134 mmol/L) Secondary to Chronic Pancreatitis Causing Critical Hyperosmolar Coma: A Case Report

**DOI:** 10.1155/crie/4737440

**Published:** 2025-06-03

**Authors:** Arnaud Robert, Sandra Ihirwe Habineza, David Leng, Chloé Dieudonné, Patrick M. Honoré, Pierre Bulpa

**Affiliations:** ^1^Intensive Care Unit, Mont-Godinne University Hospital, Yvoir, Belgium; ^2^Emergency Department, Mont-Godinne University Hospital, Yvoir, Belgium

**Keywords:** case report, diabetes mellitus, hyperosmolality, hyperosmolar hyperglycemic state, insulin therapy, intensive care

## Abstract

Hyperosmolar hyperglycemic state (HHS) is a life-threatening condition characterized by extreme hyperglycemia, high plasma osmolality, and severe dehydration without significant ketoacidosis. Prompt diagnosis and appropriate management are essential to reduce morbidity and mortality, which range from 10% to 20%. We report a case of a 50-year-old man with insulin-dependent diabetes mellitus secondary to chronic alcoholic pancreatitis presenting with severe HHS and coma. His initial blood glucose level was 134 mmol/L (2420 mg/dL), and serum osmolality was 416 mOsm/kg. Despite the critical condition at admission, the patient responded well to intensive therapy, including insulin infusion and intravenous fluids, and could be discharged without any neurological sequelae.

## 1. Introduction

Hyperosmolar hyperglycemic state (HHS) is a severe complication of diabetes mellitus, most commonly observed in elderly patients with type 2 diabetes. HHS is characterized by extreme hyperglycemia (typically >33.3 mmol/L (>600 mg/dL), total serum hyperosmolality (>320 mOsm/kg), and profound dehydration [1]. Unlike diabetic ketoacidosis (DKA), HHS lacks significant ketosis and acidosis [1, 2] (pH > 7.30 and absence of significant ketonemia [1]). Mortality rates can be as high as 20% [1–3], particularly in older patients presenting with profound hyperosmolality, higher plasma glucose, altered mental status, infection, renal impairment, or in those with delayed treatment [3–5]. The pathophysiology involves a relative insulin deficiency that is still sufficient to prevent lipolysis [6, 7] and ketosis [7, 8] but allows severe hyperglycemia to develop, leading to osmotic diuresis, dehydration, and hyperosmolality [2, 7–9]. Due to the lack of randomized studies, HHS management is largely adapted from DKA protocols, although outcomes tend to be less favorable [2]. Although cerebral edema is rare in the adult population (<1%), it may significantly exacerbate the clinical course and contribute to increased morbidity and mortality [10], especially in marked hyperglycemia and severe osmolality. The cornerstone of treatment involves rehydration, cautious correction of hyperglycemia and other electrolyte imbalances, and addressing precipitating factors, such as infection, nonadherence to therapy, new-onset diabetes, or other causes, such as alcohol and substance use, pancreatitis, pulmonary embolism, or myocardial infarction [1, 8].

## 2. Case Report

A 50-year-old male was found unresponsive at home (last seen well 16 h earlier) and was brought to the emergency department for altered mental status. On arrival, physical examination revealed hypotension (96/45 mmHg), bradycardia (heart rate: 56 bpm), hypoxemia (oxygen saturation: 88%), and hypothermia (temperature: 35.1°C). He demonstrated signs of severe dehydration and was comatose (Glasgow Coma Scale (GCS) score of 8/15), requiring endotracheal intubation and mechanical ventilation.

His medical history included: a history of drug abuse (cocaine), depression, exocrine pancreatic insufficiency, and uncomplicated insulin-dependent diabetes mellitus secondary to chronic pancreatitis due to alcohol consumption. His diabetes was managed in the long term with insulin analogs using a basal/bolus regimen (long-acting analogs of 15 U and 6–15 units of ultrarapid analogs per meal) for the past 10 years. It remained poorly controlled as indicated by his glycated hemoglobin level of 14.2%. He had no known chronic complication related to his diabetes, although he was known for diet, treatment, and follow-up nonadherence. His body weight was 60 kg with a BMI of 17.9 kg/m^2^.

Initial laboratory data revealed an extreme hyperglycemia of 134 mmol/L (2420 mg/dL), a serum sodium of 128 mEq/L due to pseudo-hyponatremia (corrected natremia of 166 mmol/L) [11], serum potassium of 4.4 mEq/L, and a bicarbonate level of 33 mEq/L. The serum osmolality was measured at 416 mOsm. He suffered from acute kidney injury with kidney disease improving global outcome (KDIGO) 2 classification [12]. There was no significant ketosis detected at the dipstick urinalysis (strictly negative ketonuria). Arterial blood gases showed a pH of 7.39. All of the above criteria (hyperglycemia, hyperosmolarity, and absence of ketosis and/or metabolic acidosis) are consistent with the diagnosis of HHS. Remarkably, his creatine kinase level was normal (60 UI/L), and he showed no sign of infection (afebrile, C-reactive protein < 10 mg/L). Chest X-ray was unremarkable. Normal cerebral CT scan appearance excluded many other causes of altered mental status. We did not find any other cause for HHS than known treatment nonadherence. Further medical history revealed that the patient had lost his subcutaneous continuous glucose monitor (e.g., Freestyle Libre) and consequently stopped administering insulin.

The patient was initially managed in the emergency department where he was treated according to our local protocol [9, 13–15] with appropriate intravenous fluid resuscitation with 0.9% saline (2.5 L in the first 5 h, 20 mL/kg of fluids the first hour) then 250 mL of fluid per hour until correction of the dehydration and was started on insulin infusion 3 h after the initiation of fluid resuscitation, at a rate of 0.05 units/kg/h. Potassium levels were monitored closely and supplemented as needed [1].

**Figure 1:**
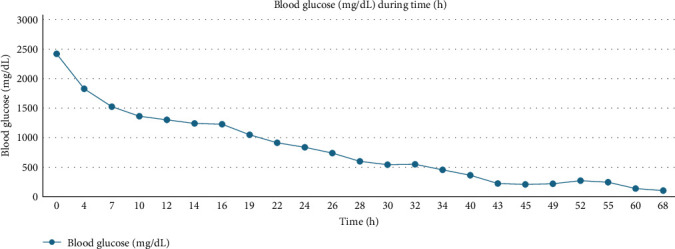
Evolution of blood glucose during time in ICU.

The objective was to reduce glycemia at a rate of 2.8–4.2 mmol/L (50–75 mg/dL) per hour; however, within 4 h, his glycemia had already dropped to 101.1 mmol/L (1829 mg/dL), and within the next 7 h, his osmolality had improved to 389. He was then transferred to the intensive care unit (ICU), where he remained on mechanical ventilation for 3 days. During that period of time, his neurological status improved as within 48 h, his blood glucose level had returned to normal (Figure 1). He received a total of approximately 12 L of fluid during the first 2 days and a total of 53.5 units of intravenous insulin for HHS to resolve. Administration of fluids and insulin is summarized in Table 1.

His ICU stay was complicated by pneumonia caused by *Streptococcus Pneumoniae*, likely secondary to broncho-aspiration, and was treated with amoxicillin–clavulanate. Mild electrolyte imbalances, including hypernatremia and hypophosphatemia, were also observed and managed effectively (Table 2). His renal function fully recovered within 48 h.

The patient was extubated on the third day and demonstrated complete neurological recovery with no signs of cerebral edema. He was discharged from the ICU after 5 days. Subcutaneous insulin was started on day 3 after ICU admission, using a basal/bolus regimen adjusted to a lower dosage (due to mild hypoglycemia) from his previous regimen. He continued to receive subcutaneous insulin therapy during his hospital stay.

After 7 days, the patient was discharged home in stable condition. He continued his insulin regimen initiated during his hospital stay and was provided a new subcutaneous continuous glucose monitor (e.g., Freestyle Libre). Follow-up consultations were scheduled to ensure appropriate glycemic control and to educate the patient on managing his diabetes to prevent future acute complications.

## 3. Discussion

This case highlights the potential for a positive outcome even in patients presenting with extremely high blood glucose levels and hyperosmolality, as seen in HHS, as mortality can be seriously affected by initial blood glucose level [4] and osmolality [16]. The patient's initial serum osmolality of 416 mOsm/kg and blood glucose of 134 mmol/L (2420 mg/dL) are among the highest reported in the literature [17], underscoring the severity of his condition. Even if rare, in such cases, the prognosis is largely determined by the risk of severe cerebral edema that can poorly affect outcome (up to 40% mortality compared to 1% if no cerebral edema occurs [10]). Managing patients with severe HHS is challenging, as it requires providing appropriate care while carefully avoiding interventions that could compromise neurological outcomes. Preventing cerebral edema remains one of the cornerstones of treatment [10, 18, 19].

The management of HHS requires a multifaceted approach, focusing on initial aggressive rehydration to restaure volemia, cautious correction of hyperglycemia, and close monitoring of electrolytes and neurological status. Early recognition and prompt initiation of treatment are crucial for preventing irreversible complications, such as cerebral edema and multiorgan failure [1, 18, 19].

In this case, the patient's satisfactory recovery can be attributed to timely ICU care, including delaying insulin therapy after appropriate fluid rescusitation [15, 19, 20] and the use of insulin infusion at a lower rate than previously recommended (0.05 U/kg/h [1, 15, 20]) (as some guidelines in the literature recommended 0.1 U/kg/h [9, 14] and sometimes, a bolus infusion of 0.14 U/kg) resulting in slower decrease in glycemia. Delaying insulin therapy after initial fluid resuscitation may be crucial in these patients because their blood volume is highly dependent on the osmotic effect of glucose levels. If insulin is initiated too early without prior adequate fluid resuscitation to restore intravascular volume depletion, it may lead to catastrophic hemodynamic collapse [19]. 2022 ISPAD guidelines and 2023 updated guidelines from the Joint British Diabetes Society recommend that insulin infusion start at least 1 h after starting fluid replacement therapy [19, 20]. The modest insulin requirement in our patient was likely due to high insulin sensitivity resulting from the etiology of his diabetes, which was secondary to chronic pancreatitis.

**Figure 2:**
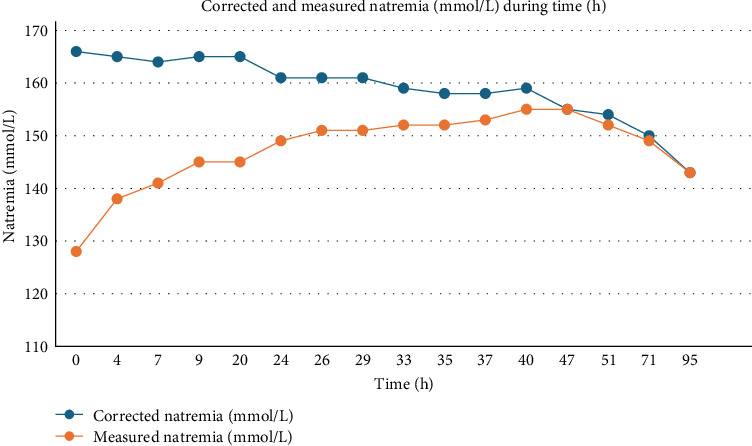
Evolution of corrected and measured natremia over time.

Similarly, the prevention of cerebral edema during hyperglycemic crisis lies in the slow decrease in blood glucose and osmolality, and also requires close monitoring of natremia during correction of glycemia. Brain cells have been demonstrated to be partially permeable to glucose because of a selective transport protein and are more sensitive to abrupt changes in natremia [21]. In HHS, cerebral edema could be due to the sudden decrease in serum sodium and blood glucose, causing a rapid drop in serum osmolality, leading to rapid fluid shift from the extracellular space toward the intracellular space, provoking brain cells swelling. This phenomenon could occur in cases of overly-aggressive fluid resuscitation, resulting in an abrupt change in serum sodium, or if fluid resuscitation is provided with hypotonic fluids. Additionally, cerebral edema may also occur when insulin is infused too rapidly, causing glucose correction at a rate that is faster than the change in serum sodium [18] while initial drop in osmolality and blood glucose level is mostly related to volume expansion and not initial insulin infusion (as illustrated in our patient with a drop from 134 mmol/L (2420 mg/dL) to 101.1 mmol/L (1829 mg/dL) solely from fluid resuscitation). In this context, and as shown in our patient, it has been demonstrated that slow correction in osmolality (3–8 mOsm/kg/h) associated with an increase in natremia appears to be a protective factor for preventing cerebral edema [18, 19]. Nevertheless, the osmotic theory for cerebral edema has been challenged by Glaser et al. [22], in a randomized study of DKA in children, where they compared two different strategies for volume expansion, showing no difference in the occurrence of subclinical cerebral edema. Their data were more consistent with vasogenic edema. Our patient's evolution in natremia is provided in Figure 2.

Additional strategies employed that may improve outcome in these situations involve lower hourly target rate of decreasing blood glucose levels, close monitoring of IV fluids administration, and as recommended by the literature, keeping the blood glucose at a steady state of 14–17 mmol/L (250–300 mg/dL) until neurological improvement [9], to prevent hypoglycemia which is well-established risk factor for increased short- and long-term mortality in critically ill patients in the initial phase [23].

Despite the severe hyperosmolar state, the patient's outcome was favorable, with no residual neurological deficits. This case underscores that, with appropriate and timely intervention, even patients presenting with extreme hyperosmolality can recover good outcomes.

## 4. Conclusion

This case report demonstrates that successful outcomes could be achieved in patients with severe HHS, even when presenting with critically high blood glucose levels and osmolality. Timely intervention and comprehensive ICU care based on the physiopathology of HHS were the key components of this patient's recovery.

## Figures and Tables

**Table 1:** Fluid administration and insulin over time during ICU stay.

Time (day)	1	2	3	4	5	Total during ICU stay
Fluid (mL)	6455	5485	4385	3150	1745	21220
Insulin (units)	27.5	26	44	39	28	164.5

**Table 2:** Table of initial laboratory workup during ICU stay.

Time (h)	0	4	07	16	40	64	88	112	136
Hemoglobin (g/dL)	15.9	—	14.7	12.6	12.5	10.4	10.1	10.3	8.4
Hematocrit (%)	49	—	43	36	37	32	31	31	28
White blood cells (cells/mm³)	18,290	—	15,870	14,040	10,980	13,720	9490	7850	11,310
Sodium (mmol/L)	128	—	141	145	155	149	143	142	142
Potassium (mmol/L)	4.4	—	3.7	4.0	3.9	3.5	3.7	3.5	3.9
Chloride (mmol/L)	78	—	94	106	120	116	115	113	112
Measured osmolality (mOsm/kg)	416	—	389	—	—	—	—	—	294
Calculated total osmolality (mOsm/kg)	—	—	—	360	330	313	297	291	—
Creatinine (mg/dL)	1.38	—	1.54	1.98	1.24	0.98	0.73	0.68	0.64
Phosphorus (mmol/L)	2.19	—	1.62	1.14	1.19	0.91	0.62	0.66	1.09
Magnesium (mmol/L)	1.46	—	1.52	1.61	1.43	1.22	0.83	0.74	0.81
Albumin (g/dL)	44.4	—	37.9	—	—	—	22.9	23.2	22.0
Glucose (mg/dL)	2420	1829	1524	1227	362	144	113	66	112

## Data Availability

The data that support the findings of this study are available from the corresponding author upon reasonable request.
